# Vaccinia virus GLV-1h153 is a novel agent for detection and effective local control of positive surgical margins for breast cancer

**DOI:** 10.1186/bcr3404

**Published:** 2013-03-18

**Authors:** Sepideh Gholami, Chun-Hao Chen, Laurence J Belin, Emil Lou, Sho Fujisawa, Caroline Antonacci, Amanda Carew, Nanhai G Chen, Marina De Brot, Pat B Zanzonico, Aladar A Szalay, Yuman Fong

**Affiliations:** 1 Department of Surgery, Memorial Sloan-Kettering Cancer Center, 1275 York Avenue, New York, NY 10065, USA; 2 Department of Medicine, Division of Hematology, Oncology, and Transplantation, University of Minnesota, 420 Delaware Street SE, Minneapolis, MN 55455, USA; 3 Molecular Cytology Core Facility, Memorial Sloan-Kettering Cancer Center, 1275 York Avenue, New York, NY 10065, USA; 4 Department of Radiation Oncology, Rebecca & John Moores Comprehensive Cancer Center, University of California, 9500 Gilman Drive, San Diego, CA 92093, USA; 5 Genelux Corporation, 3030 Bunker Hill Street, San Diego, CA 92109, USA; 6 Departments of Medical Physics and Radiology, Memorial Sloan-Kettering Cancer Center, 1275 York Avenue, New York, NY 10065, USA; 7 Rudolf Virchow Center for Experimental Biomedicine, Department of Biochemistry and Institute for Molecular Infection Biology, University of Würzburg, Sanderring 2, Würzburg, 97074, Germany

## Abstract

**Introduction:**

Surgery is currently the definitive treatment for early-stage breast cancer. However, the rate of positive surgical margins remains unacceptably high. The human sodium iodide symporter (hNIS) is a naturally occurring protein in human thyroid tissue, which enables cells to concentrate radionuclides. The hNIS has been exploited to image and treat thyroid cancer. We therefore investigated the potential of a novel oncolytic vaccinia virus GLV1h-153 engineered to express the hNIS gene for identifying positive surgical margins after tumor resection via positron emission tomography (PET). Furthermore, we studied its role as an adjuvant therapeutic agent in achieving local control of remaining tumors in an orthotopic breast cancer model.

**Methods:**

GLV-1h153, a replication-competent vaccinia virus, was tested against breast cancer cell lines at various multiplicities of infection (MOIs). Cytotoxicity and viral replication were determined. Mammary fat pad tumors were generated in athymic nude mice. To determine the utility of GLV-1h153 in identifying positive surgical margins, 90% of the mammary fat pad tumors were surgically resected and subsequently injected with GLV-1h153 or phosphate buffered saline (PBS) in the surgical wound. Serial Focus 120 microPET images were obtained six hours post-tail vein injection of approximately 600 μCi of ^124^I-iodide.

**Results:**

Viral infectivity, measured by green fluorescent protein (GFP) expression, was time- and concentration-dependent. All cell lines showed less than 10% of cell survival five days after treatment at an MOI of 5. GLV-1h153 replicated efficiently in all cell lines with a peak titer of 27 million viral plaque forming units (PFU) ( <10,000-fold increase from the initial viral dose ) by Day 4. Administration of GLV-1h153 into the surgical wound allowed positive surgical margins to be identified via PET scanning. *In vivo*, mean volume of infected surgically resected residual tumors four weeks after treatment was 14 mm^3 ^versus 168 mm^3 ^in untreated controls (*P *< 0.05).

**Conclusions:**

This is the first study to our knowledge to demonstrate a novel vaccinia virus carrying hNIS as an imaging tool in identifying positive surgical margins of breast cancers in an orthotopic murine model. Moreover, our results suggest that GLV-1h153 is a promising therapeutic agent in achieving local control for positive surgical margins in resected breast tumors.

## Introduction

Surgery is currently the definitive treatment for early-stage breast cancer. However, the rate of positive surgical margins remains unacceptably high. Current techniques for detecting positive margins include frozen sectioning, touch prep cytology, gross evaluation, intra-operative imaging with ultrasonography, and conventional histopathology. Most meta-analyses report a positive margin rate of 20 to 80% with current techniques. One report showed that intra-operative assessment of gross margins resulted in a 25% final margin positive rate [1]. Another study from investigators at MD Anderson showed that a combination of intra-operative gross margin assessment, specimen radiography and frozen section still resulted in a final positive margin rate of 20% [2]. Other groups have compared radio-guided occult lesion localization with wire localization, yielding high positive margin rates of 83% and 56%, respectively [3]. All current techniques have limitations and to our knowledge there are no data to support a statement that any strategy is superior to another. Overall, the literature still demonstrates that up to 40% of the patients return to the operating table for a re-excision despite the best efforts for breast-conserving surgery [4].

Oncolytic viral therapies have shown great success in preclinical and clinical testing as a novel cancer treatment modality [5]. Oncolytic viral therapy relies on the ability of genetically engineered viruses to specifically infect, replicate within and ultimately lyse tumor cells [6]. Specifically, Yu *et al. *showed via low light and fluorescence imaging how vaccinia virus gained entry and replicated only in the tumor tissue without causing viremia in live animals [7]. Moreover, the safety of using vaccinia viruses in humans has been well demonstrated during the eradication of smallpox virus [8].

Recently, a novel vaccinia virus, GLV-1h153, has been shown to be simultaneously safe, diagnostic and therapeutic for the use against several human cancers, including anaplastic thyroid and pancreatic cancer in animal models [9,10]. Here, we report the potential of this vaccinia virus carrying the human sodium iodide symporter (hNIS) in identifying positive surgical margins in an orthotopic breast cancer model. Moreover, we tested whether GLV-1h153 can be used as adjuvant therapy for positive margins of breast cancer and to provide local control of residual tumors.

## Materials and methods

### Cell lines

Human TNBC cell lines HCC38 and MDA-MB-468 were obtained from the ATCC (American Type Culture Collection, Manassas, VA USA). Human MDA-MB-231 breast carcinoma cells stably expressing mCherry fluorescent protein were kindly provided by Dr. Koblinski from Northwestern University. HCC38 cells were maintained in RPMI (supplemented with Hepes, L-glutamine, sodium pyruvate, final concentrations 10, 2, 1 mM, respectively, and with 1.5 g/L sodium bicarbonate, 4.5 g/L glucose). MDA-MB-231 and MDA-MB-468 cells were maintained in Dulbecco's modified Eagle's medium (DMEM)/F12 containing 5% FBS, 100 IU/mL penicillin/streptomycin, 1 mM sodium pyruvate, 2X nonessential amino acids. Medium for MDA-MB-231 mCherry cells was supplemented with 1 μg/mL blasticidin (Sigma-Aldrich, St. Louis, MO USA}). CV-1 cells for viral plaque assay were maintained in Minimum Essential Medium (MEM). All media were supplemented with 10% fetal calf serum with penicillin (100,000 U/L) and streptomycin (100 mg/L). All cell lines were grown in a 5% CO_2 _humidified incubator at 37°C.

### Virus

GLV-1h153 is a replication-competent, recombinant vaccinia virus based on the vaccinia virus LIVP strain (Lister strain from the Institute for Research on Virus Preparations, Moscow, Russia) and its construction has been described previously [10]. Pertinent to our study, GLV-1h153 expresses hNIS and GFP. The genotype of hNIS-expressing VACV GLV-1h153 was verified by PCR and sequencing.

### Green fluorescent protein expression

Cells were plated at 2 × 10^4 ^per well in 96-well plates in 200 μL of media. After overnight incubation, cells were exposed to GLV-1h153 at a multiplicity of infection (MOI) of 0.1, 1.0 or 5.0. Cells were examined using fluorescence-inverted microscopy (Nikon Eclipse TE300, Nikon, Japan) to detect GFP expression at set time intervals (24, 48, 72 hours).

### Cytotoxicity assays

Cells were plated at 3 × 10^4 ^cells per well in 24-well plates and incubated overnight. GLV-1h153 was added to each well at varying MOIs of 0, 0.1, 1.0 and 5.0. Viral cytotoxicity was tested using a lactate dehydrogenase (LDH) assay daily. Cells were washed with phosphate buffered saline (PBS) once and then lysed with Triton X-100 (1.35%, Sigma, St. Louis, MO, USA). The intracellular LDH release following lysis was subsequently measured with a Cytotox 96 kit (Promega, Madison, WI, USA) on a spectrophotometer (EL321e, Bio-Tek Instruments, Winooski, VT USA}) at 490 nm. Results were expressed as the percentage of surviving cells, calculated as the LDH release of infected samples compared to negative control (cells without virus). All samples were analyzed in triplicate.

### Viral plaque assay

After infection of cells with virus and prior to daily cytotoxicity assay, supernatants of each infected well were collected daily for five days and immediately frozen at -80°C for storage. Serial dilutions of each supernatant samples were made to perform standard viral plaque assays on confluent CV-1 culture plates. All samples were measured in triplicate and the results averaged.

### Xenograft model and surgical resection

All mice were cared for and maintained in accordance with animal welfare regulations under a protocol approved by the Institutional Animal Care and Use Committee (IACUC) of Memorial Sloan-Kettering Cancer Center (MSKCC). Orthotopic mammary fat pad xenografts were generated in female athymic nude mice aged 8 to 10 weeks. Mice were anesthetized with 1 to 3% isoflurane and injected with 100 μL of 5 × 10^6 ^MDA-MB-231 mCherry-expressing cells suspended in 50% matrigel (BD Biosciences, Franklin Lakes, NJ, USA) into the mammary fat pad. After two weeks of tumor formation, 90% of each tumor was surgically resected from the mammary fat pad so that only a small margin of tumor (< 5 mm in the largest dimension) remained. Subsequently, GLV-1h153 (1 × 10^6 ^PFU)/50 μL PBS) or 50 μL of PBS for controls were injected into the surgical wound and the wound was closed with a stapler.

### *In vivo * fluorescent imaging (Maestro)

*In vivo *fluorescent images were obtained with the CRi Maestro system (Cambridge Research and Instrumentation, Woburn, MA, USA) using the appropriate filter set (excitation = 575 to 605 nm, emission = 645 nm long-pass filter). After each image was obtained, it was spectrally unmixed to remove the background fluorescence and overlay images were produced.

### *In vivo * PET imaging

Control and GLV1h153-treated groups of three animals bearing MDA-MB-231 tagged with mCherry orthotopic mammary fat pad tumors were injected with 600 μCi of ^124^I via tail vein injection four days after surgical resection. Six hours after radiotracer administration, three-dimensional list-mode data were acquired. Imaging was performed using a Focus 120 microPET dedicated small animal PET scanner (Concorde Microsystems Inc, Knoxville, TN, USA). The count rates in the reconstructed images were converted to activity concentration (%ID/g) using a system calibration factor (MB q/mL per cps/voxel) derived from imaging of a mouse size phantom filled with a uniform aqueous solution of ^18^F. Image analysis was performed using ASIPro software (Siemns Medical Solutions USA, Inc. Malvern, PA USA).

### Treatment of positive surgical margin in an orthotopic TNBC xenograft model

A total of 14 female athymic nude mice (Harlan Laboratories, Indianapolis, IN USA) aged six to eight weeks were injected with 5 × 10^6 ^MDA-MB-231-mCherry cells unilaterally into the fourth mammary fat pad. Four weeks after tumor inoculation, surgical resection of tumors was performed (as described above) and mice were randomized into two groups. Xenografts were treated with GLV-1h153 (1 × 10^6 ^PFU/50 μL PBS) or 50 μL of PBS for controls into the surgical wound at the time of resection. The size of the residual tumor created at the positive margins (length and width) was recorded immediately after resection and one month after treatment with GLV-1h153. Tumor volumes were calculated by the equation, V (mm^3^) = (4/3)*(π)*((a/2)^2^*(b/2)) where 'a' is the smallest diameter and 'b' the largest diameter. Four weeks after treatment, mice were sacrificed and tumors were harvested and embedded in paraffin for histological analysis.

### Microscopic imaging

A Lumar V.12 stereo-microscope (Carl-Zeiss Oberkochen, Germany) with 1.2x objective was used to image surgically resected tumors that were excised from the animal at the end of the study using bright field and fluorescent techniques.

### Fixation, tissue preparation and H&E staining

To prepare tissue sections for H&E staining, tissues were fixed with 4% paraformaldehyde in PBS overnight at 4°C, washed in 70% ethanol and processed for paraffin embedding per the standard protocol of the Molecular Cytology Core Facility of MSKCC. Five-micron sections were cut serially from paraffin-embedded tumor tissue blocks. Slides were air dried and baked at 60°C for one hour. We confirmed that all margins were evaluated histologically by a board-certified pathologist at MSKCC.

### Statistical analysis

All results were reported as means with standard errors. The significance of differences between different groups was determined using the Student's *t *test (Excel 2007; Microsoft, Redmond, WA, USA). A *P*-value < 0.05 was considered significant.

## Results

### GLV-1h153 demonstrated time- and dose-dependent infectivity *in * cell cultures

**Figure 1:**
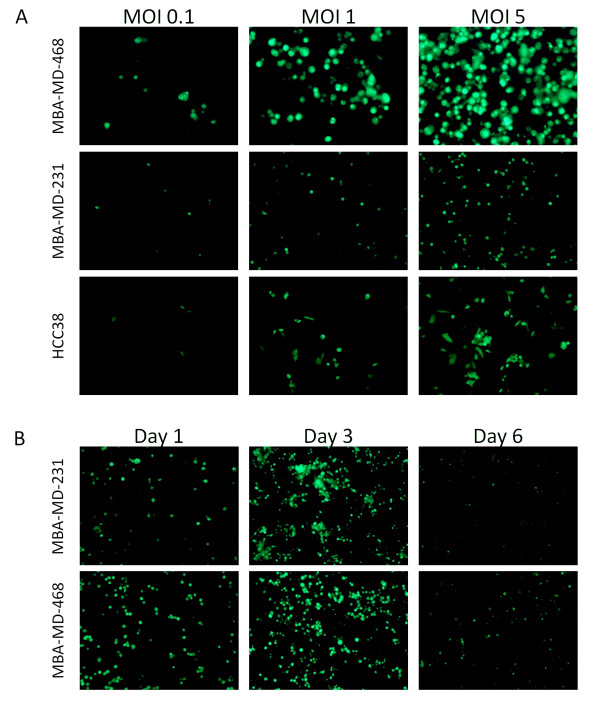
**Cell lines MDA-MB-468, MDA-MB-231 and HCC38 show sensitivity to infection with GLV-1h153**. **A**. Viral infection was concentration-dependent as shown by increasing number of GFP-expressing cells. **B**. Viral infection demonstrated to be time-dependent. GFP expression increased early after infection as shown from Day 1 to Day 3, but then decreased when cell death occurred (by Day 6). Illustrated were images from infection at an MOI of 1.

Viral infectivity and GFP expression were assessed 24, 36 and 72 hours after viral infection at an MOI of 0.1, 1 or 5 by fluorescence microscopy (Figure 1A). GFP expression confirmed viral infection by 24 hours in all cell lines and was proportional to viral concentration. As depicted in Figure 1B, viral infection was time-dependent, increasing from Day 1 to Day 3 and decreasing by Day 6 as cell death occurred in all cell lines.

### GLV-1h153 induced cell death in a dose-dependent fashion in TNBC lines *in vitro *

**Figure 2:**
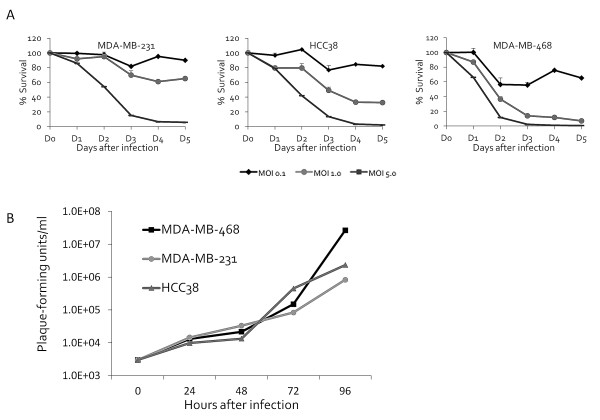
**GLV-1h153 kills and replicated efficiently in breast cancer cell lines *in vitro***. **A**. GLV-1h153 kills breast cancer cells in a dose-dependent fashion. Cytotoxicity data of cell lines MDA-MB-231, MDA-MB-468 and HCC38 showed less than 10% viable cells after five days of infection at an MOI of 5. Multiplicity of infection (MOI) = plaque-forming units/cell. Experiment was performed in triplicates. **B**. GLV-1h153 replicated efficiently in all breast cancer cell lines. All cell lines supported viral replication as assessed by the viral plaque assay. Results demonstrated that in cell line MDA-MB-468, GLV-1h153 reached the highest titer of 2.7 × 107 PFU after 96 hours of infection, representing over a 10,000-fold increase in copy numbers from the initial viral dose. The experiment was performed in triplicates.

GLV-1h153 killed all TNBC cell lines effectively in a dose-dependent fashion (Figure 2A). At an MOI of 5, GLV-1h153 achieved near-complete cell kill in all cell lines by Day 5. At lower viral concentrations (MOI 1), there was still significant viral potency to MDA-MB-468 and HCC38 with greater than 92% and 67% cytotoxicity effect, respectively. Survival curves indicate time-dependent cell kill in all cell lines as expected from the results of the viral infectivity assay and GFP expression.

### GLV-1h153 replicates efficiently in all TNBC cell lines

Standard viral plaque assays were performed to assess for viral replication in TNBC cells. After infection with GLV-1h153 at an MOI of 1, all TNBC cell lines allowed efficient viral replication (Figure 2B). For all cell lines, the viral titer exhibited up to a 4-log, or 10,000-fold, increase from the initial viral dose only 96 hours after infection. MDA-MB-468 supported the highest viral titer, with a peak titer of 2.7 × 10^7\^PFU/mL four days after infection.

### *In vivo * visualization of positive surgical margins with fluorescent imaging

**Figure 3:**
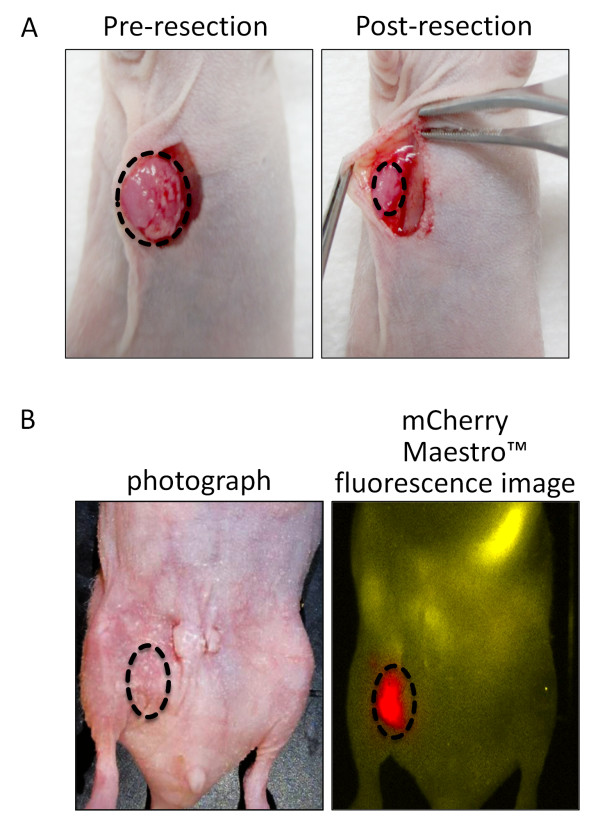
**Surgical margins of breast cancer after resection can be detected via mCherry fluorescence imaging *in vivo***. **A**. Photographs of an animal pre- and post-surgical resection of the mammary fat pad tumor shown in upper panel. Tumor is outlined by dotted line. **B**. Bottom panel shows a photograph after surgical resection and infection with a closed wound. To the right is shown a mCherry Maestro fluorescence image to confirm that a positive margin was present prior to PET imaging.

Ninety percent of the tumors were surgically resected from the mammary fat pad, so that only a small remnant of tumor (< 5 mm in the largest dimension) remained (Figure 3A). To ensure that a positive margin was present after surgical resection of the mammary fat pad tumors, mCherry expression was assessed four days later by Maestro™ fluorescence imaging (PerkinElmer, Waltham, MA USA). *In vivo *fluorescence imaging demonstrated minimal mCherry signal localized to the surgical margin of the xenografts in all surgically resected tumors (Figure 3B).

### GLV-1h153 identifies positive surgical margins via hNIS-mediated radioiodine uptake by PET

**Figure 4:**
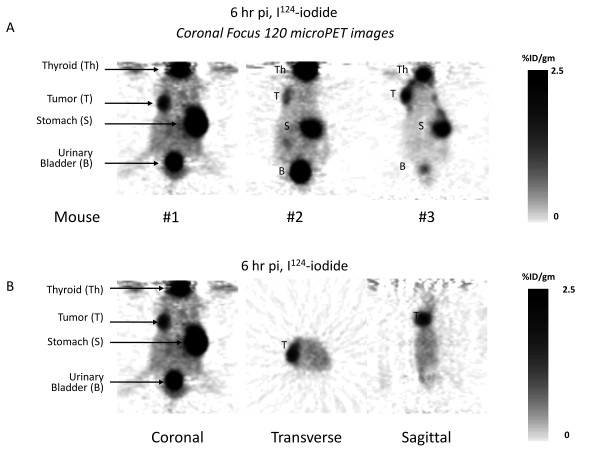
**GLV-1h153 can identify positive surgical margins of breast cancer after resection via hNIS-mediated radio-uptake**. **A**. microPET images shown six hours after radiotracer injection of all three infected animals after surgical resection. **B**. Three-dimensional (3D) view of PET images of one infected animal at the surgical margin.

*In vivo*, positive surgical margins of infected mammary fat pad tumor wounds treated at the time of resection with GLV-1h153 were successfully visualized by PET imaging. All GLV-1h153-injected animals' wounds showed accumulation of ^124^I-iodine radioactivity localized to the tumors (Figure 4A) compared with uninfected controls. Positive margins were visualized as early as six hours after radiotracer injection in all three infected animals. Average radioiodine uptake in the 3 GLV-1h153-infected mice with positive margins was 2.5 ± 0.30% ID/g six hours post radiotracer injection.

### GLV-1h153 effectively prevents progression of residual tumor at surgical margins *in vivo *

**Figure 5:**
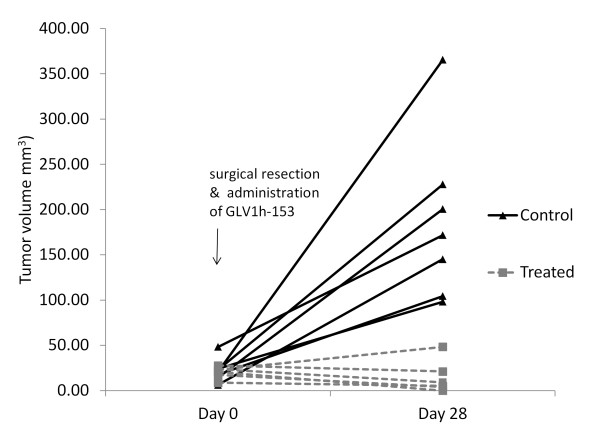
**GLV-1h153 effectively prevents progression of residual breast tumor at surgical margins *in vivo***. Residual tumors at surgical margins of control mice grew from an average size of 25 mm^3 ^to 169 mm^3 ^(over a four-fold increase in tumor volume) compared to the average tumor size of treated mice which regressed from 20 mm^3 ^to 14 mm^3 ^in infected surgical margins within four weeks of treatment after resection (*P *< 0.05). Three out of six of the treated mice demonstrated complete regression of residual tumor.

*In vivo*, positive surgical margins of mammary fat pad tumor were treated at the time of resection with GLV-1h153 or PBS. Infected residual tumors were successfully controlled with viral therapy while uninfected control tumors continued to grow over the course of a month after tumor resection. GLV-1h153-injected positive margins averaged a size of 14 mm^3 ^four weeks after treatment compared to 168 mm^3 ^in uninfected controls (Figure 5). All mice tolerated treatment without any complications or side effects. Fifty percent (three out of the six) of treated mice demonstrated complete regression of residual tumor. H&E staining of the three remaining treated margins showed necrosis and fibrosis and only a few viable tumor cells present. Sections of uninfected positive margins, in contrast, demonstrated invasive carcinoma of high histologic grade associated with lymphovascular invasion as shown by tumor emboli within the dermal lymphatics. Tumor cells exhibited a high degree of nuclear pleomorphism with large nuclei, irregular chromatin and conspicuous nucleoli.

### GLV1h153 can be detected in GPF-expressing cells in positive surgical margins

**Figure 6:**
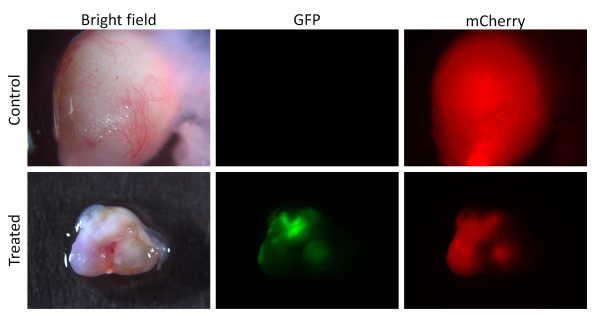
**GPF-expressing malignant cells are detected in positive surgical margins with fluorescent microscopy**. Uninfected control tumors (treated with PBS) express mCherry only and no GFP signal (top panel). Bottom panel represents an infected remaining tumor margin four weeks after treatment with GLV-1h153. The tumor expresses mCherry as well as GPF signal.

**Figure 7:**
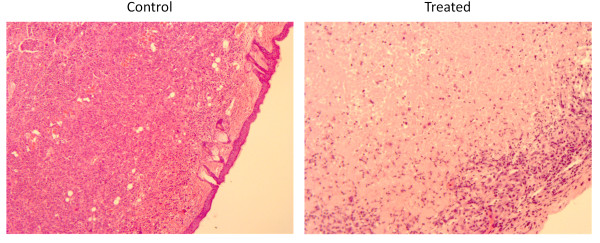
**H&E slides of untreated and treated positive surgical margins**. Positive-margin controls (left) show malignant cells with large nuclei and irregular chromatin. Treated positive margins H&E slides (right) represent tissue margins four weeks after treatment with GLV-1h153. Large areas of fibrosis and necrosis can be seen without any viable cancer cells.

Four weeks after viral infection with GLV-1h153, resected surgical margins were imaged to confirm if viral particles could be detected within tumor margins. GFP-positive cells were visible within all three remaining tumors at the surgical margin after treatment. Virus-infected tumor margins both expressed mCherry and GFP in contrast to uninfected tumors, which only expressed mCherry (Figure 6). H&E staining of tissues from control tumors showed malignant cells consistent with TNBC compared to large areas of fibrosis and necrosis in GLV-1h153-treated tumors without any viable cancer cells (Figure 7).

## Discussion

Surgical resection is currently the mainstay of treatment for early-stage breast cancer, with the goal of obtaining negative surgical margins. With the advent and acceptance of breast-conserving therapy and radiation, the definition of a safe surgical margin and the indications and extent of re-excision have been the subject of frequent debate and study. A 20-year follow-up prospective randomized clinical trial conducted by the National Surgical Adjuvant Breast and Bowel project demonstrated equivalent overall and disease-free survival in the treatment of early stage breast cancer by mastectomy compared to breast conservation therapy and radiation. However, high rates of positive surgical margins ranging from 20 to 80% remain a major issue surrounding conservative resection [11]. It is now widely accepted in the literature that obtaining negative surgical margins is an important predictor for local recurrence after wide excision of invasive breast cancer [12-15]. Moreover, studies have shown that patients with positive surgical margins have a higher risk of distant failure after five years [16,17]. While wider margins of resection increase the likelihood of obtaining negative margins, it comes at an increasing financial, cosmetic and psychological cost to the patient. Furthermore, obtaining wide margins is not always feasible if a prior excision leaves no residual breast tissue behind. The challenge is to remove enough breast tissue to prevent recurrence, while saving an adequate breast volume to yield satisfactory cosmetic results.

Presently the intra-operative assessment of margins include gross examination of the specimen by the operating surgeon and the pathologist for residual tumor, ultrasound, radiography of the specimen, frozen section, touch prep cytology and sampling of tumor cavity margins. Unfortunately, all current methods to determine margin status have some technical or practical limitation [1,18]. Thus, innovative techniques of identifying positive surgical margins are needed to decrease the high rate of re-operation for patients with breast cancer.

The historical use of vaccinia as a vaccine for smallpox demonstrated its safety when it was given to millions of people, with a very low toxicity rate of less than 1%. Moreover, specific deletions within the genome of oncolytic viruses allow for more specific tumor targeting. In the case of GLV-1h153, inactivation of the thymidine kinase (TK) gene, for example, makes the replication more dependent on host cell TK for deoxyribonucleotide synthesis, which may allow for increased selectivity of viral replication for malignant neoplasms [12,13]. Second, the TK mutation may attenuate the ability of vaccinia to replicate within normal tissues, making it safer for clinical application [14]. Other anti-apoptotic genes, such *SPI-1 *and *SPI-2 *[16], have also been shown to enable preferential viral replication selectively in tumor tissues, allowing for more targeted therapy. In our studies, we showed how vaccinia specifically targets tumors without affecting the surrounding organs. For example, when the parent virus (GLV-1h68) was used to treat anaplastic thyroid cancer xenografts *in vivo*, virus biodistribution in mice 10 days after a single viral intratumoral injection showed high viral recovery in tumors, but only minimal recovery in other organs [19].

For our *in vitro *and xenograft model, we chose to work with a TNBC model. While this work is relevant to the treatment and imaging of receptor-positive breast cancer as well, TNBCs represent a subset of invasive breast cancers whose lack of receptor targets for hormonal therapy confer an even greater dependency upon complete, margin-free surgical resection for patient survival. In our study, we demonstrated the ability of a new oncolytic vaccinia virus engineered to express hNIS to identify positive surgical margins of resected mammary fat pad tumors in an orthotopic breast cancer model via I^124^-iodine PET imaging while providing concurrent local control to residual tumors.

*In vivo*, mammary fat pad tumors generated from mCherry-tagged MDA-MB-231 cells that were incompletely resected and treated with GLV-1h153 prior to wound closure resulted in visualization of the residual tumor margin by I^124^-iodine PET scanning. Uninfected controls did not show any evidence of radiotracer accumulation and could not be imaged via PET imaging. This was a proof-of-concept study with the purpose of assessing the ability of the virus to serve as an effective visible marker of a positive surgical margin. To adequately mimic resection of early-stage human malignancy, this model will eventually need to be applied to microscopic margins. Other research groups have used different viral vectors, such as adenovirus, to increase hNIS expression and allow for radionuclide uptake in prostate and medullary thyroid cancer [20-22]. But to our knowledge, this is the first study to show increase in hNIS expression and accumulation of radioiodine in a TNBC model. One of the largest studies showed that approximately 70% of breast cancers (hormone receptor positive as well as TNBC) [23] express hNIS, but smaller series have only shown no greater than 34% hNIS expression [24]. Such an expression level, however, has not been proven to be sufficient for therapeutic radioiodine accumulation.

In our study, all animals infected with GLV-1h153 increased the expression of hNIS, required to concentrate radioactive iodine for imaging. Moreover, our study showed that GLV-1h153 can directly act as an adjuvant therapeutic agent and treat residual tumors at the surgical margin identified on imaging. Our results demonstrated a significant difference in tumor volume between infected and uninfected residual tumors four weeks after a single virus injection into the surgical wound. In addition, in remaining infected margins, the GFP signal was still present by fluorescence microscopy four weeks after treatment. This result has two important implications. First, detection of GFP in cells indicates active viral infection and thus possible continued treatment. Second, the presence of GFP signal can also be used as a diagnostic tool in the clinical setting in detection of residual disease. Altogether, the utility of this novel virus in the present work is its ability to provide early and direct visualization of residual tumor in the resection bed while simultaneously treating the remnant tumor cells. In addition, PET imaging may allow for noninvasive detection of viral distribution. The tracking of viral delivery could give clinicians the ability to correlate therapeutic efficacy and monitor potential viral toxicity. In the clinical setting, one potential approach of the virus would be used in a two-step procedure: the virus would be applied to the surgical wound at the time of resection followed by multiple post-resection I^124^-iodine PET scans (at two- to four-month intervals). The expected I^124^-iodine tumor uptake would provide a metric of residual disease post-resection and the difference in uptake between subsequent scans would give a metric of therapeutic response. Therefore, GLV-1h153 can be utilized potentially as a therapeutic agent and predictor of therapeutic response.

There are both technical and biological limitations of the method proposed for detection of microscopic and residual disease. The former include PET scanner sensitivity and, most importantly, resolution. The latter include specific and non-specific uptake of radioiodide. As detailed above, and as largely determined by the spatial resolution of the scanner, the minimum number of cells detectable by PET in a cluster is approximately 100 million. Low tumor-specific uptake of the radiotracer and non-specific uptake by normal tissues decrease the tumor-to-normal tissue ratio and thus further degrade the detectability of microscopic/residual disease. For example, significant uptake of fluorine-18-labeled fluorodeoxyglucose (FDG), the most widely used oncologic PET tracer, occurs in non-malignant inflammatory cells, such as macrophages and neutrophils, reducing the specificity of FDG PET for malignant disease. An important potential advantage of our method is improved specificity. Radioiodide is significantly concentrated only in the thyroid, salivary glands, stomach and lactating breast; it is otherwise rapidly cleared from the body. GLV1h153 treatment plus I^124^-iodine thus has the potential to yield highly specific as well as sensitive PET images of malignant disease.

## Conclusions

In conclusion, this pilot study demonstrates that a mutated vaccinia virus expressing hNIS can identify a ositive surgical margin in an orthotopic breast cancer model and effectively treat remaining tumors *in vivo*. Positive surgical margins remain a major issue despite best current efforts. Therefore, our data strongly encourage the continued investigation of GLV-1h153 as a novel imaging tool for identifying positive surgical margins and adjuvant therapeutic agents in the treatment of breast cancer.

## Abbreviations

DMEM: Dulbecco's modified Eagle's medium; FDG: fluorodeoxyglucose; GPF: green fluorescent protein; hNIS: human sodium iodine symporter; LDH: lactate dehydrogenase; MEM: Minimum Essential Medium; MOI: multiplicity of infection; MSKCC: Memorial Sloan-Kettering Cancer Center; PBS: phosphate buffered saline; PET: Positron Emission Tomography; PFUs: plaque-forming units; TK: thymidine kinase; TNBC: Trple-negative breast cancer; VACV: vaccinia virus.

## Competing interests

Yuman Fong is a scientific consultant to Covidien, Ethicon, Genentech, and Genelux; Nanhai Chen and Aladar Szalay are employees of Genelux Corporation; Sepideh Gholami, Chun-Hao Chen, Laurence Belin, Emil Lou, Sho Fujisawa, Caroline Antonacci, Amanda Carew, Marina De Brot, and Pat Zanzonico declare no competing interests. Genelux Corporation has applied for a patent on hNIS expressing viruses.

## Authors' contributions

SG and CC designed the entire study together, carried out all major experiments with the help of others and primarily drafted the manuscript. EL and LB helped with the design of the study and helped write the manuscript. NG helped with the design of the viral construct and its production, as well as with writing the manuscript. CA and AC helped with all the *in vitro *assays (infection, LDH and viral plaque assays). SF helped with microscopy imaging and finalizing figures. MB performed the path review. PZ helped with PET imaging and analysis of imaging data. YF conceived of the study, and participated in its design and helped to draft the manuscript. All authors read and approved the final manuscript.
